# Construction of pH-responsive and up-conversion luminescent NaYF_4_:Yb^3+^/Er^3+^@SiO_2_@PMAA nanocomposite for colon targeted drug delivery

**DOI:** 10.1038/srep21335

**Published:** 2016-02-19

**Authors:** Boshi Tian, Shaohua Liu, Wei Lu, Lin Jin, Qingfeng Li, Yurong Shi, Chunyang Li, Zhenling Wang, Yaping Du

**Affiliations:** 1 The Key Laboratory of Rare Earth Functional Materials and Applications, Zhoukou Normal University, Zhoukou 466001, P. R. China; 2 University Research Facility in Materials Characterization and Device Fabrication, The Hong Kong Polytechnic University, Hong Kong, P. R. China; 3 Frontier Institute of Science and Technology Jointly with College of Science, Xi’an Jiaotong University, Xi’an 710049, P. R. China

## Abstract

Colon-targeted drug delivery system has attracted much interest because it can improve therapeutic efficacy and reduce the side effect in practical clinic. Herein, we constructed a multifunctional drug delivery system with colonic targeting and tracking by up-conversion (UC) luminescence based on core-shell structured NaYF_4_:Yb^3+^/Er^3+^@SiO_2_@PMAA nanocomposite. The resultant materials exhibited bright UC luminescence, pH-responsive property and excellent biocompatibility. The drug release behaviors in different pH environment were investigated using 5-aminosalicylic acid (5-ASA) as a model drug. The 5-ASA molecules release from NaYF_4_:Yb^3+^/Er^3+^@SiO_2_@PMAA nanocomposite exhibit a significant pH-responsive colon targeted property, i.e., a little amount of drug release in simulated gastric fluid (SGF, pH = 1.2) but a large amount of drug release in simulated colonic fluid (SCF, pH = 7.4) Moreover, the drug release process could be monitored by the change of UC emission intensity. These results implied that the multifunctional nanocomposite is a promising drug carrier for targeted release of 5-ASA in the colon.

5-Aminosalicylic acid (5-ASA) is a typical anti-inflammatory drug, which is commonly used in the treatment of inflammatory bowel disease (IBD). Unfortunately, most of orally administered 5-ASA molecules are likely to be absorbed in the stomach (pH = 1–2) before reaching the colon sites (pH = 7–8), causing the therapy efficiency to reduce drastically^1^,^2^,^3^. To overcome this shortcoming, many efforts have been devoted to constructing oral colon targeted drug delivery system for the treatment of IBD in recent years^4^,^5^,^6^,^7^,^8^. The drug delivery system has gained much attention because it has many advantages over the traditional drug treatment, such as protecting the drug in the strong acid conditions (e.g. in the stomach), precisely releasing the drug at the targeted site, improving the therapy efficiency, reducing the side effects on non-target site, *etc*. In recent years, colon targeted drug delivery system based on various materials, such as inorganic nanostructures^9^, polymers^10^,^11^ and chitosan^12^, has attracted much attention due to that it can intelligently transport drugs to the targeted tissue by the stimulation of physiopathological pH signal. As a typically pH-responsive hydrogel, poly (methacrylic acid) (PMAA) is widely used as drug carriers and can be swollen/collapsed at a certain pH, which arises from the deionization/ionization of –COOH groups (pKa = 4~5) in PMAA^13^,^14^. At the lower pH (below the pKa) such as in acidic stomach condition, the PMAA polymer can maintain a collapsed state and thus reduce the loaded drug leakage. While at the higher pH (above the pKa) such as in colonic condition, the PMAA networks are in a swollen state because the –COOH groups are ionized as –COO^−^ and then repelled each other, thereby releasing the encapsulated drugs to medium^13^,^14^,^15^,^16^. Additionally, PMAA polymer is generally recognized as good biocompatibility and biodegradability, so it is a very suitable drug carrier for target release drugs in the colon conditions^13^,^14^.

Lanthanide doped up-conversion nanoparticles (UCNPs), which can convert light of long wavelength (typically near-infrared) into shorter-wavelength (mostly visible) luminescence via the multi-photon process, have received increasing attention in recent years^17^,^18^,^19^,^20^,^21^,^22^,^23^,^24^,^25^. Compared with other luminescent materials, such as organic dyes and quantum dots, UCNPs have many advantages including a weak autofluorescence background, excellent photostability and chemical stability, low toxicity and high tissue penetration depth in biological tissues when excited with a near-infrared light (NIR) source^26^,^27^,^28^,^29^,^30^. Moreover, UCNPs can be used as drug carrier for tracking and evaluation of the drug release in living system^31^,^32^. Such outstanding features make UCNPs be suitably used in biomedical applications such as drug delivery carrier^26^,^33^,^34^,^35^,^36^, magnetic resonance imaging (MRI)^37^,^38^, bioimaging^39^,^40^,^41^, *etc*.

If one could combine the merits of a smart pH-responsive polymer and the UCNPs to fabricate a multifunctional nanocomposite and be used for drug carrier, it would be have a potential application in targeted drug delivery system. The pH-responsive polymer coated on the surface of UCNPs have been proved to exhibit a charge switching property in different pH conditions for pH-responsive drug release^36^. Thus, the nanocarrier not only can transport drug to the target site but also can track the process of drug release. Up to now, many multifunctional nanocomposites consisted of smart polymer and magnetic nanoparticles^8^,^42^,^43^,^44^,^45^ or mesoporous silica^6^,^46^, have been synthesized. For example, Chen and co-workers fabricated a nanocomposite by coating thermo- and pH-responsive polymer on the surface of Au@SiO_2_ and studied the target drug release property *in vivo*^47^. Lin’s group designed a multifunctional nanocomposite through filling smart polymer into the cavity of down-conversion luminescent GdVO_4_ hollow spheres and this nanocomposite can be used for pH-controlled release of anti-cancer drug doxorubicin hydrochloride (DOX)^38^. Subsequently, they developed a multifunctional nanocarrier based on UCNPs coated with thermo/pH-coupling sensitive polymer poly[(*N*-isopropylacrylamide)-co-(methacrylic acid)] as shell for tumor targeted release of DOX^26^,^28^. At pH = 7.4, a small quality of DOX release because the polymer shells keep swollen. At pH = 5.0, the polymer shells collapse and become hydrophobic, leading to the DOX was squeezed out the polymer shells. However, up to now, there is no report about the combination of UCNPs with stimuli-responsive polymer to design a pH-responsive drug delivery system which can targeted release of drug in SCF for treatment of IBD.

Herein, we designed a novel pH-sensitive colon targeted drug delivery system by grafting PMAA polymer onto the silica coated UC luminescent NaYF_4_:Yb^3+^/Er^3+^ nanospheres (UCNPs), wherein PMAA was used as shell to load and control release of drug 5-ASA, and UCNPs were utilized as optical probe to monitor the drug release process. Our results demonstrated that 5-ASA can be efficiently loaded on the multifunctional nanocarrier and 5-ASA could be targeted release in colonic condition (pH = 7.4), indicating that the multifunctional nanocomposite have promising prospect in colon targeted oral drug delivery systems.

## Results

**Figure 1:**
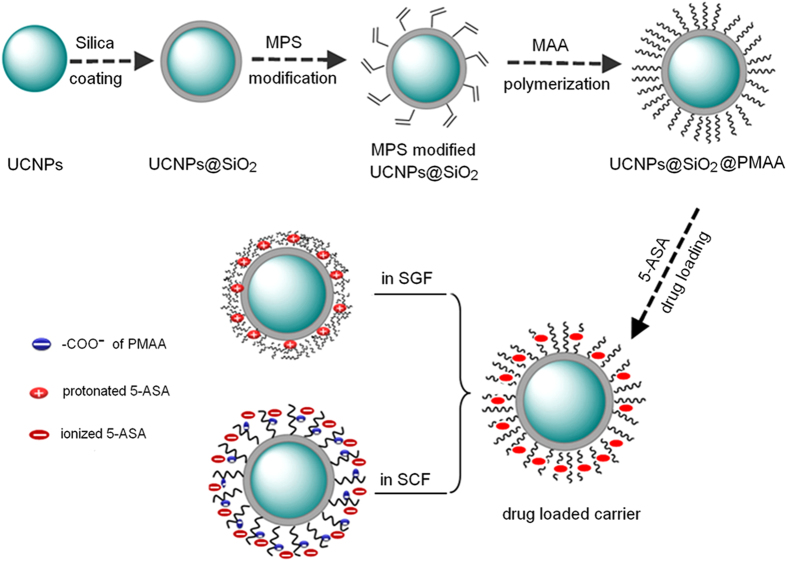
Schematic illustration of the preparation procedure of UCNPs@SiO_2_@PMAA nanocomposite and controlled release of 5-ASA.

In this study, a novel pH-responsive and up-conversion luminescent NaYF_4_:Yb^3+^/Er^3+^@SiO_2_@PMAA nanocomposite was fabricated for targeted release of 5-ASA. As depicted in Fig. 1, firstly, the α-NaYF_4_:Yb^3+^/Er^3+^ nanoparticles (UCNPs) were coated with a layer of solid silica via a simple *sol-gel* process. Next, the UCNPs@SiO_2_ nanospheres were modified with MPS, which have a C = C bond and could react with the monomers in the polymerization. Then, the UCNPs@SiO_2_-MPS was copolymerized with monomer MAA in the aqueous solution to get the final UCNPs@SiO_2_@PMAA nanocomposite. Finally, 5-ASA was chosen as a model drug to investigate the drug release behaviours in SCF and SGF, respectively. Expectedly, 5-ASA can be confined to the collapsed PMAA network to avoid releasing ahead when pass through the stomach and targeted releasing in the colon.

### Formation of UCNPs@SiO_2_@PMAA nanocomposite

**Figure 2:**
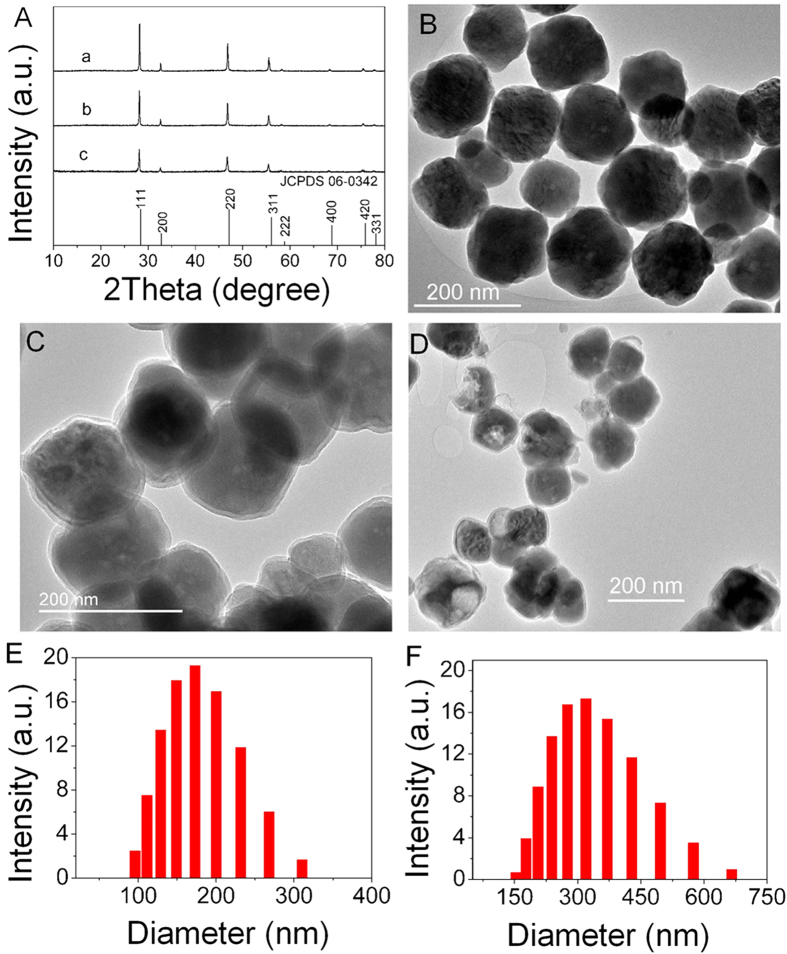
(**A**) XRD patterns of UCNPs (a), UCNPs@SiO_2_ (b) and UCNPs@SiO_2_@PMAA (c). TEM images of UCNPs (**B**), UCNPs@SiO_2_ (**C**) and UCNPs@SiO_2_@PMAA (**D**). Histograms of the size distributions of UCNPs@SiO_2_ (**E**) and UCNPs@SiO_2_@PMAA (**F**).

Figure 2A shows the XRD patterns of UCNPs, UCNPs@SiO_2_ and UCNPs@SiO_2_@PMAA. For UCNPs (Fig. 2A-a), all intense peaks can be well indexed to cubic phase of α-NaYF_4_ (JCPDS No. 06-0342, space group: *Fm-3m*, Z = 2). Additionally, no peaks corresponding to any other phases or impurities were detected, indicating the high purity of UCNPs samples. For UCNPs@SiO_2_ (Fig. 2A-b), the position of diffraction peaks is not changed compared to that of the UCNPs, indicating that the UCNPs@SiO_2_ still possess cubic structure. However, the intensity decreases slightly due to the silica coating on the surface of UCNPs^48^. After PMAA polymer was grafted on the surface of UCNPs@SiO_2_ (Fig. 2A-c), the diffraction intensity is further decreased. This might be due to the reduction in the electron density of the samples after coated with PMAA polymer.

The morphology and microstructure of samples were examined by TEM. From the Fig. 2B, we can observe that the UCNPs consisted of well dispersed nanospheres with an average diameter of about 120 ± 20 nm. As can be seen from the TEM and HRTEM images (Fig. S1(A–C), Supplementary Information), the obtained UCNPs show a well-resolved lattice fringes and the calculated distance between the adjacent lattice fringes is about 0.32 nm. The selected area electrical diffraction (SAED) pattern (Fig. S1(D)) indicates that the UCNPs possess a polycrystal structure. The energy dispersive X-ray spectroscopy (EDS) confirms the presence of yttrium (Y), ytterbium (Yb), erbium (Er), sodium (Na) and fluorine (F) in the UCNPs sample (Fig. S1(E)). It can be seen from the TEM image of UCNPs@SiO_2_ (Fig. 2C) that the UCNPs nanospheres can be successfully coated with uniform gray silica shell (~8 nm) and the spherical morphology can be still retained. After PMAA grafted on the surface of UCNPs@SiO_2_ (Fig. 2D), the morphology of UCNPs@SiO_2_@PMAA has little change except for poor dispersibility compared with UCNPs@SiO_2_, indicating the PMAA polymerization process has no distinct effects on the morphology of UCNPs@SiO_2_ nanospheres. However, the polymer coating PMAA on the surface of UCNPs@SiO_2_ was not observed form the TEM image, probably due to the weak constrast between the polymer and the UCNPs@SiO_2_ nanospheres.

Additionally, the EDS results (Fig. S2) show that the C element exists in the UCNPs@SiO_2_@PMAA nanocomposite due to the coating of PMAA polymer. Moreover, the hydrodynamic diameter of UCNPs@SiO_2_ is about 173 nm (Fig. 2E), which is larger than that observed from TEM images because of the presence of hydrate layer in an aqueous environment. After being grafted of PMAA, the hydrodynamic diameter of UCNPs@SiO_2_@PMAA added up to about 324 nm (Fig. 2F).

**Figure 3:**
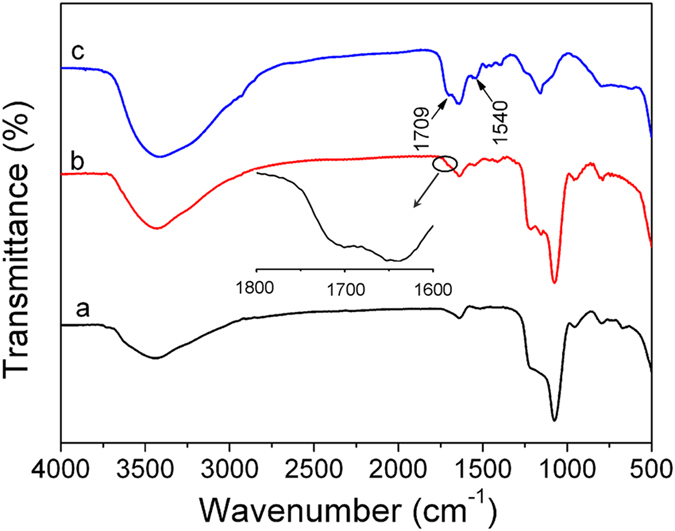
FT-IR spectra of the UCNPs@SiO_2_ (a), UCNPs@SiO_2_-MPS (b) and UCNPs@SiO_2_@PMAA (c). The inset shows enlargement of the selected IR absorption bands in the range from 1600 to 1800 cm^−1^.

The surface modification of UCNPs@SiO_2_ with MPS and further coating with PMAA polymer was investigated by FT-IR spectroscopy. For UCNPs@SiO_2_ (Fig. 3a), the absorption bands at 3450 cm^−1^ (OH), 1630 cm^−1^ (H_2_O), 1080 cm^−1^ (*ν*_*s*_: Si-O-Si), 800 cm^−1^ (*ν*_*as*_: Si-O-Si) and 965 cm^−1^ (*ν*_*s*_: Si-OH) are present^49^,^50^. After modification with MPS (Fig. 3b), a new absorption band at 1705 cm^−1^ can be observed and is assigned to the vibration of C = O from MPS, suggesting that the UCNPs@SiO_2_ nanospheres were successfully functionalized with MPS. For the UCNPs@SiO_2_@PMAA (Fig. 3c), the characteristic of PMAA absorption band (C = O: 1709 cm^−1^) was clearly observed, while the bands at 2928 cm^−1^ and 1540 cm^−1^ could be assigned to the stretching vibration absorption of C-H and asymmetric stretching vibration absorption of COO^−^ anion groups from PMAA, which confirmed that the PMAA can be grafted onto the surface of the UCNPs@SiO_2_ nanospheres^43^,^51^. These results further confirm that the coating of PMAA was successful in our experimental conditions. To determine the quantitative amount of the PMAA content grafted onto the surface of UCNPs@SiO_2_, the TGA measurement was performed. As shown in Fig. S3, the PMAA content in the nanocomposite is about 13 wt.% from the TG curves in the range of 100–800 °C.

### UC luminescence and cytotoxicity of nanocomposite

**Figure 4:**
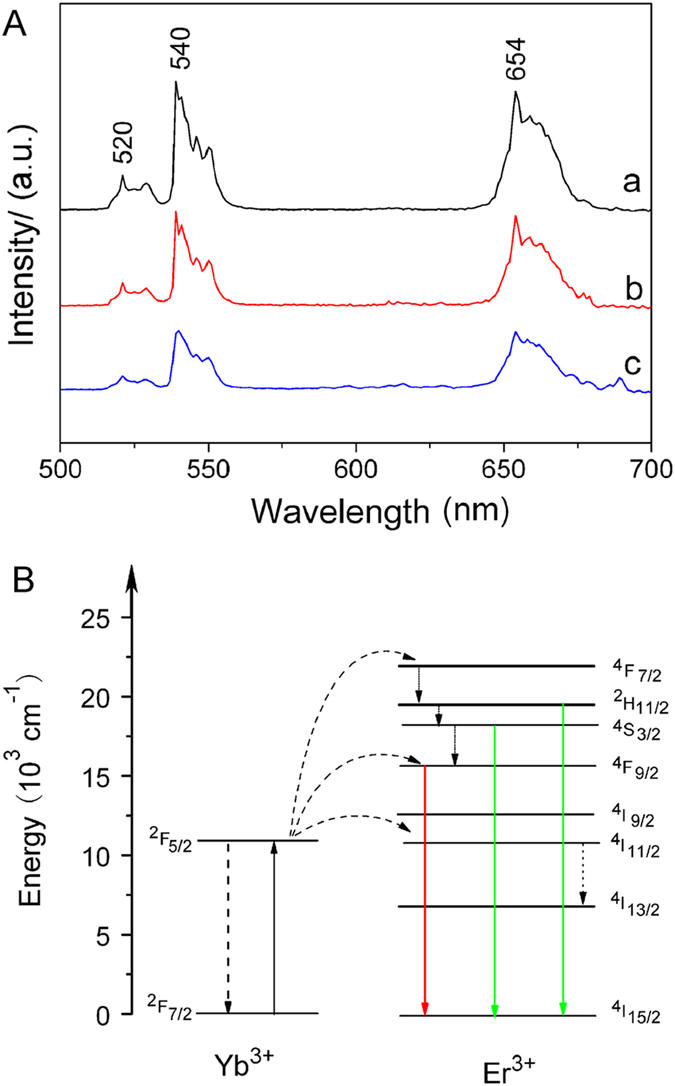
(**A**) UC emission spectra of UCNPs (a), UCNPs@SiO_2_ (b) and UCNPs@SiO_2_@PMAA (c). (**B**) Schematic energy levels of Yb^3+^ and Er^3+^ and the UC luminescence process excited by 980 nm laser.

The UC luminescence spectra of UCNPs, UCNPs@SiO_2_ and UCNPs@SiO_2_@PMAA samples under 980 nm excitation are displayed in Fig. 4A. Compared with UCNPs, the other samples exhibit similar emissions except for the slightly decrease of intensity, suggesting that the silica shell and polymer on the surface of UCNPs did not significantly affect the UC properties. The spectra of these three samples exhibit two green peaks at about 520–540 nm and a red peak at about 654 nm, which are assigned to energy transitions from ^2^H_11/2_ → ^4^I_15/2_, ^4^S_3/2_ → ^4^I_15/2_ and ^4^F_9/2_ → ^4^I_15/2_ of Er^3+^ ions, respectively. The UC fluorescence mechanism of Er^3+^, Yb^3+^ co-doped nanocomposite is shown in Fig. 4B. Yb^3+^ initially absorbs a 980 nm NIR light and subsequently transferred the energy to a nearby Er^3+^ ion, exciting Er^3+^ to the ^4^I_11/2_ level. Then a second 980 nm photon by the excited Yb^3+^ can populate the ^4^F_7/2_ level of Er^3+^, afterward Er^3+^ will relax nonradiatively to the ^2^H_11/2_ and ^4^S_3/2_ levels. Finally resulting in the green (520 nm, ^2^H_11/2_ → ^4^I_15/2_; 540 nm, ^4^S_3/2_ → ^4^I_15/2_) and red (654 nm, ^4^F_9/2_ → ^4^I_15/2_) emission, respectively.

**Figure 5:**
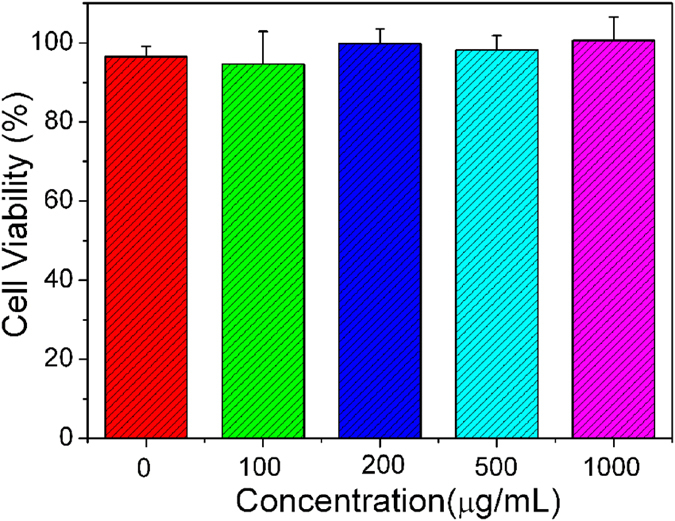
Cell viability of hMSCs after incubation with UCNPs@SiO_2_@PMAA nanocomposite for 24 h and quantitative assays by MTT assay.

It is well known that a drug carrier used in biomedical fields for controlled release of drugs must be nontoxic. Therefore, the cytotoxicity of the synthesized nanocomposite was evaluated using a MTT assay performed on hMSCs. As shown in Fig. 5, more than 94.9% of viabilities can be observed over a varying concentration range (from 100 to 1000 μg/mL), indicating the UCNPs@SiO_2_@PMAA nanocomposite has no obvious cytotoxicity on hMSCs. The above results indicate that the nanocomposite has good biocompatibility as drug carrier in biomedical application.

### Targeted drug release and release kinetics

**Figure 6:**
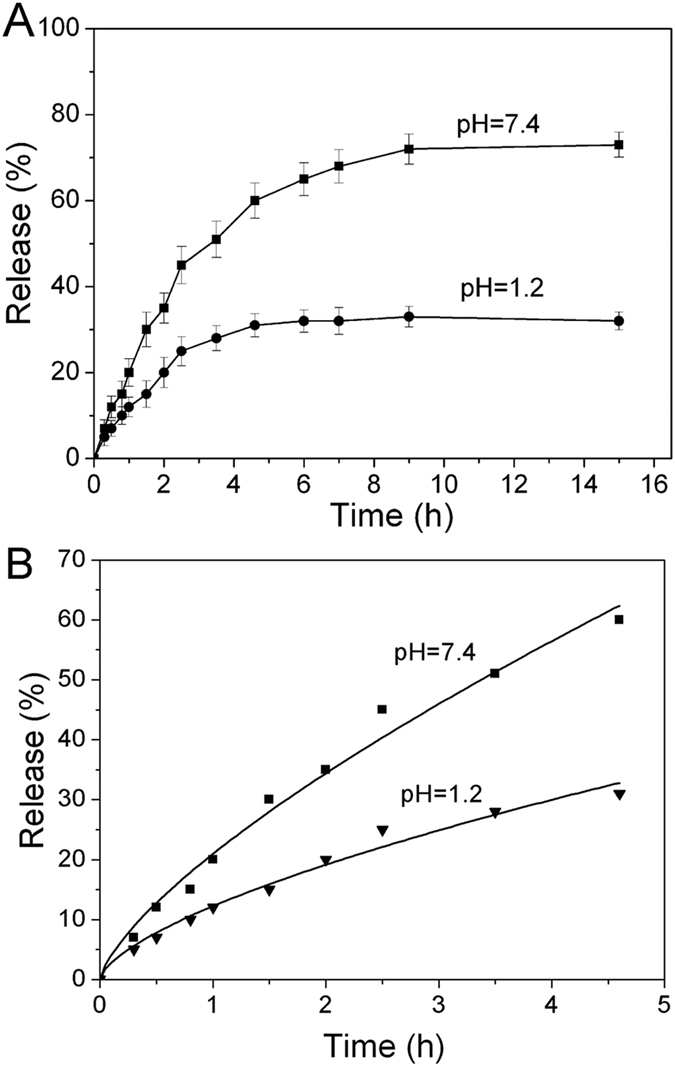
(**A**) Release profiles and (**B**) Ritger-Peppas plot of 5-ASA from UCNPs@SiO_2_@PMAA nanocomposite in SCF (pH 7.4) and in SGF (pH 1.2), respectively.

Indeed, a colon targeted drug delivery systems require that the drug exhibits no or little release before reach the colon site, otherwise, the drug will be adsorbed ahead, resulting in therapy efficiency decrease and side effects. The drug release profiles from UCNPs@SiO_2_@PMAA nanocomposite in simulated colonic fluid (SCF, pH = 7.4) and simulated gastric fluid (SGF, pH = 1.2) are displayed in Fig. 6A. As can be seen, the release rate and percentage of drug 5-ASA strongly depend on the value of pH. At pH = 1.2 in SGF, the release amount of 5-ASA was less than 20% during the first 2 hours (stomach transmit time), while at pH = 7.4 in SCF, the release amount reached 35%. The total cumulative amount of drug release in pH = 7.4 SCF was up to about 70% after 10 hours, which was 2.5 times higher than that in pH = 1.2 SGF (about 30%), suggesting the carrier can transport the 5-ASA molecules pass through the stomach with little release and mainly release in the colon. To further figure out the progress of drug release, the release data were also analyzed by kinetic models. The obtained UCNPs@SiO_2_@PMAA nanocomposite, as a swellable spherical carrier, its release behavior can be described according to the Ritger-Peppas model (equation 1)^52^,^53^.


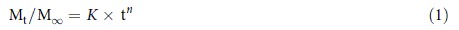


where M_t_/M_∞_ is the cumulative drug release amount at time t. *K* is a constant, which is related to the structure of the carrier, *n* is the release exponent characterizing the diffusion mechanism. It has been confirmed that the values of *n* = 0.45, 0.43 < *n* < 0.85 and n = 0.85 indicate Fick diffusion (case I), non-Fick (anomalous) transport, and diffusion and zero-order transport (case II) mechanism, respectively^52^,^53^,^54^. Drug release data in SCF and SGF solutions were analyzed by the equation (1). The fitting results were presented in Table 1 and Fig. 6B.

As can be seen from Table 1, the obtained correlation coefficient (*R*^2^) is 0.99 and 0.98, respectively, indicating drug release data fit well to the Ritger-Peppas model both in SCF and SGF solutions. The calculated *n* values were between 0.43 and 0.85 in both cases, suggesting that the drug diffusion mechanism belongs to the non-typical Fick transport, which regarded as the superposition of both Fick diffusion and swelling controlled drug release^54^,^55^. Furthermore, the results in Table 1 showed that *K* values at pH = 7.4 (about 20.9) is much higher than that at pH = 1.2 (about 12.2), suggesting the drug release faster in SCF solution (pH = 7.4) than in SGF solution (pH = 1.2)^56^,^57^. This coincide with the results shown in Fig. 6A.

### Mechanism of drug release

**Figure 7:**
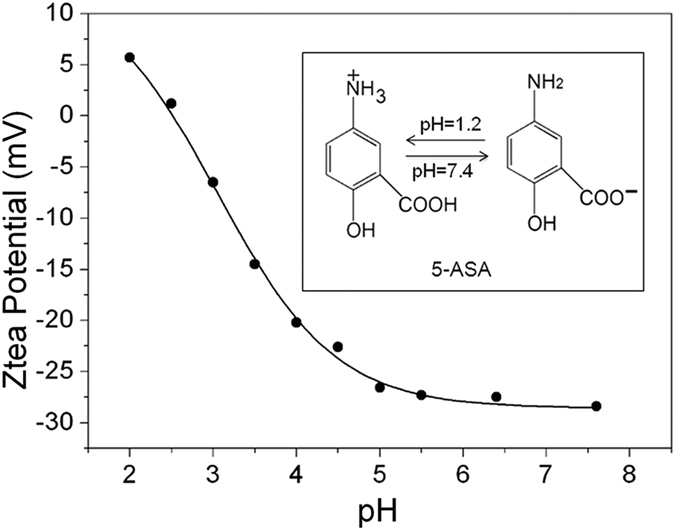
The ζ-potential of UCNPs@SiO_2_@PMAA and (inset) ionized 5-ASA molecular at different pH values.

To make clear the mechanism of drug release is vital to further study the drug delivery system. The pH-responsivity of UCNPs@SiO_2_@PMAA nanocomposite is derived from the polymer PMAA grafted on surface of carrier. Figure 7 shows the ζ-potential of UCNPs@SiO_2_@PMAA at different pH values. As depicted in Fig. 7, under lower pH condition, especially less than 2.5, the nanocomposite shows a positive charge arising from the protonated carboxylic acid groups. In contrast, under alkaline condition, the ζ-potential values decrease to about −28 mV because the large amount of carboxylic acid groups were deprotonated as carboxylic acid anions at higher pH condition, resulting in a high negative charge density of UCNPs@SiO_2_@PMAA. Additionally, according to the Fig. 7, the p*K*a of UCNPs@SiO_2_@PMAA is estimated to be about 4~5. In the case of 5-ASA, as shown in Fig. S4, it mainly contains amino group (pKa = 5.26) and carboxylic acid group (pKa = 2.09)^55^, which can be protonated and ionized respectively under different pH conditions (see inset of Fig. 7). Actually, the degree of ionized of 5-ASA in different pH solutions can be calculated by Henderson-Hasselbalch formula^58^,^59^.

**Figure 8:**
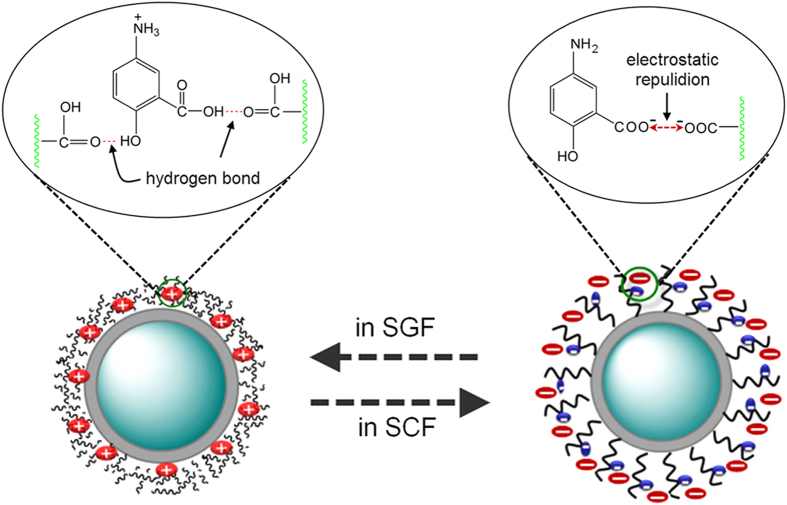
Schematic representation of pH-responsive release of 5-ASA from nanocomposite.

Previous reports showed that the drug release behaviour strongly depended on the state of polymer opened/collapsed and interaction between polymer and drug^51^,^58^. Thus, in this work, the pH-dependent drug release from nanocomposite may be explained as follows. Firstly, At a higher pH (pH = 7.4), which was above pKa of PMAA, most of the carboxylic acid groups of PMAA ionized as –COO^−^ anions and will repel each other, causing the polymer shell to be swelled and the network to be opened. By contrast, at lower pH (pH = 1.2), the polymer shell was contracted or collapsed due to the formation of hydrogen bond among the carboxylic groups of PMAA. Secondly, the intermolecular interaction between PMAA and drug has an effect on the drug diffusional process. If there exists an electrostatic attraction or intermolecular hydrogen-bonding interaction between them, it will hinder the drug diffuse through the network of PMAA. On the contrary, if there exists an electrostatic repulsion, it will accelerate the rate of diffusion of the drug. Therefore, the drug release mechanism can be described in Fig. 8 based on above discussion. In SGF solution, the PMAA network begin to shrink, resulting in most of the drug molecules can hardly be penetrated through the high-density PMAA membrane and restricted in the polymer matrix. Additionally, under such strong acid condition (pH = 1.2), 87.9% of 5-ASA molecules became protonated and formed two kinds of hydrogen bonds between the carboxylic acid groups of PMAA segment and drug 5-ASA, i.e., carboxylic acid groups of PMAA segment with carboxylic acid group and hydroxyl group of 5-ASA, respectively. Thus, such strong hydrogen bonds will also hinder the drugs diffusion out of the polymer matrix into the SGF solution^58^. So the accumulative release of 5-ASA in SGF was much lower than that of in SCF. However, it also should be noted that a fast release of 5-ASA can be observed during the initial 2 hour (Fig. 6A) in SGF, which was attributed to the drugs located on the outer surface of UCNPs@SiO_2_@PMAA nanocomposite. However, in the SCF solution, the polymer PMAA stayed a swollen state and the network opened arising from the carboxylic acid groups ionized, causing the drug molecules to diffuse out of the comparatively loose network easily. Besides, at such pH condition, 5-ASA is almost completely (about 99.1%) ionized as HOC_6_H_3_NH_2_COO^−^ anions, which has the same charges of PMAA. Consequently, as shown in Fig. 8, such strongly electrostatic repulsion and associated with the disappearance of hydrogen-bonding interaction between them will promote a fast rate release of drugs into the SCF solution^43^. From the above discussion, we can conclude that the drug release behaviour from carrier is mainly controlled by two pH dependent factors: the network of PMAA opened or closed and the interaction between drug and carrier.

### Monitoring of drug release

**Figure 9:**
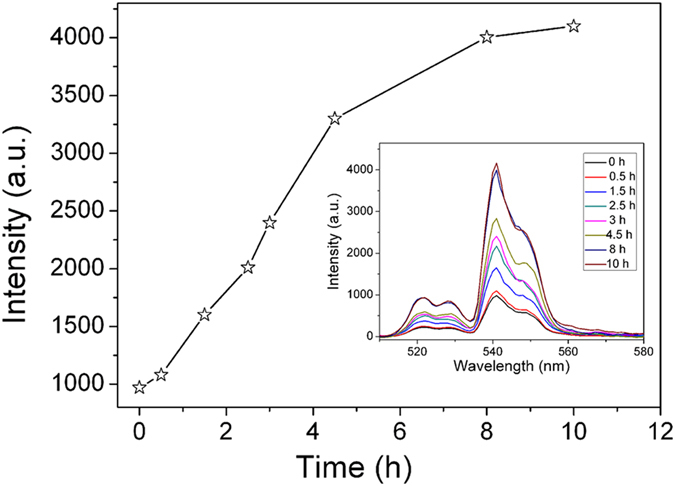
UC emission intensity of UCNPs@SiO_2_@PMAA nanocomposite as a function of release time.

To monitor the 5-ASA release process and efficiency in SCF, the relationship between UC emission intensity of 5-ASA loaded UCNPs@SiO_2_@PMAA carrier and cumulative release of 5-ASA was studied. As shown in Fig. 9, the UC emission intensity increases with the increasing of release amount of 5-ASA and keeps steady at the maximum amount of 5-ASA release, similar to the results reported by others^28^. This may arise from the 5-ASA molecules with high vibration frequencies that could quench some the luminescent centers of nanocomposite after 5-ASA loaded. With the release of 5-ASA, the quenching effect will be weakened, causing the emission intensity of nanocomposite to increase. According to previous study, UCNPs can penetrate the tissues under a 980 nm NIR light laser excitation and also can be used as luminescent probe for cell imaging^26^,^36^. Thus, in this paper, we can track the drug release process by the change of the emission intensity under the NIR light laser excitation. The relationship between the emission intensity and drug release extent can be potentially used as a probe for tracking the drug release.

## Conclusions

In summary, core-shell structured UCNPs@SiO_2_@PMAA nanocomposite consisting of UCNPs core and crosslinked PMAA shell has been synthesized and subsequently employed as a drug carrier to control the release of model drug 5-ASA. The *in vitro* cellular cytotoxicity test using the MTT assay proved that the nanocomposite is highly biocompatible and suitable for application in colon targeted drug delivery system. The drug delivery system exhibits significantly pH-dependent release of 5-ASA. The drug release behaviour showed that the cumulative release amount of 5-ASA in SCF is much higher than that in SGF, indicating that 5-ASA could be targeted delivery to the colon condition. Moreover, the mechanism of 5-ASA release from the carrier is investigated in detail, revealing that the drug release behaviour mainly depended on the state of PMAA network and the interaction between drug and carrier. Additionally, the UC emission intensity of carrier increases with the release of 5-ASA. The results demonstrate that the multifunctional nanocomposite is significantly pH-responsive and can be potentially used as stimuli sensitive carrier for oral colon targeted drug delivery.

## Methods

### Synthesis of NaYF_4_:Yb^3+^/Er^3+^ nanospheres (UCNPs)

Up-conversion NaYF_4_:Yb^3+^/Er^3+^ nanospheres (UCNPs) were prepared according to the procedure reported by Sun *et al.*^60^. The molar ratio of Ln^3+^/EDTA/NaF is 1/1/12, and the lanthanide ion concentration in the precursor is 0.04 M.

### Synthesis and surface modification of UCNPs@SiO_2_

The silica-coated UCNPs were prepared by a modified *sol-gel* method^61^. Typically, the obtained UCNPs nanospheres (100 mg) were dispersed in ethanol (80 mL) and treated with ultrasonator for 15 min. Then, distilled water (20 mL) and concentrated ammonia aqueous solution (1.5 mL, ~28 wt.%) were added. Subsequently, TEOS (50 μL) was added to the solution and stirring for 6 h at room temperature. The precipitate was collected by centrifugation, and washed with abundant water and ethanol in turn. In order to modify the surface of UCNPs@SiO_2_ nanospheres with MPS silane coupling agent, the above product was directly added into ethanol (100 mL) containing of MPS (0.5 mL). After stirring for 24 h at 25 °C, the UCNPs@SiO_2_-MPS was collected by centrifugation and washed three times with ethanol.

### Grafting PMAA on the surface of UCNPs@SiO_2_

Grafting the pH-sensitive polymer PMAA layer on the surface of UCNPs@SiO_2_ was executed by the precipitation polymerization of MAA in distilled water. Typically, the obtained MPS-modified UCNPs@SiO_2_ was redispersed in distilled water (50 mL) containing sodium lauryl benzenesulfate (5.0 mg). After stirring for 1 h, MAA monomers (200 mg) and BIS (15 mg) were added. Under the bubbling of nitrogen gas for 30 min, APS solution (2 mL, 5 mg/mL) was inject quickly, and the polymerization was carried out with stirring at 75 °C in an oil bath for 4 h. The resulting UCNPs@SiO_2_@PMAA nanocomposite was collected by centrifugation, then washed thoroughly with abundant distilled water to remove the excess reactants and any detachable polymer, and dried in vacuum at 40 °C for 24 h.

### Drug loading and release

The obtained UCNPs@SiO_2_@PMAA nanocomposite (50 mg) was dispersed into a solution of 5-ASA (1.0 mg/mL) in PBS buffer solution (10 mL) under stirring at 25 °C for 24 h, then the sample was separated by centrifugation and washed twice with PBS solution. The amount of 5-ASA adsorbed was calculated by UV-vis spectrophotometer at 298 nm (see Supplementary Fig. S4). The standard curve of 5-ASA is displayed in Fig. S5. The 5-ASA loaded UCNPs@SiO_2_@PMAA nanocomposite was dispersed into of PBS solution (20 mL, pH 1.2 or 7.4) at 37 °C with gentle shaking. At predetermined time, the suspension was centrifugated, then 2 mL of supernatant was withdrawn and replaced with equal volume of the corresponding fresh PBS to keep the volume and carrier concentration constant throughout the experiment. The amount of release 5-ASA was determined using UV-vis spectrophotometer.

### *In vitro* cytotoxicity of UCNPs@SiO_2_@PMAA nanocomposite

The *in vitro* cytotoxicity of UCNPs@SiO_2_@PMAA nanocomposite was measured by MTT assay on human mesenchymal stem cells (hMSCs). Cells with a density of 8000 cells per well were cultured in a 96-well plate in 5% CO_2_ at 37 °C for 24 h. Then different concentrations of UCNPs@SiO_2_@PMAA (100, 200, 500 and 1000 μg/mL) were added to the culture wells and subsequently incubated for 24 h at 37 °C in 5% CO_2_. Then, 20 μL of 5 mg/μL MTT (3-(4, 5-dimethyl-2-thiazolyl)-2, 5-diphenyl-2-H-tetrazolium bromide) solution was added to every well and incubated for another 4 h in 5% CO_2_ at 37 °C. After the culture medium was removed by MTT solution, 150 μL of dimethyl sulfoxide (DMSO) was added to each well and shaken for 5 min at room temperature. The optical density (OD) value of the mixture was measured at 490 nm.

### Characterization

Powder X-ray diffraction (XRD) patterns were obtained on a Bruker D8 Focus diffractometer using Cu Kα radiation (λ = 0.15406 nm). Transmission electron microscope (TEM) was performed on JEOL-2100F with a field emission gun operating at 200 kV. The hydrodynamic diameter and ζ-potential of nanoparticles were determined on Zetasizer Nano ZS90 (Malvern instrument, UK) at 25 °C. Thermogravimetric analysis (TGA) was carried out on a Perkin–Elmer STA-6000 with a heating rate of 10 °C /min in a temperature range from 30 to 800 °C under N_2_ atmosphere. Fourier transform infrared (FT-IR) spectra were recorded on a Nicolet 5700 infrared spectrophotometer (Thermo Fisher Scientific, American) using the KBr pellet technique. The up-conversion (UC) emission spectra were obtained on a FLS920P Edinburgh Analytical Instrument (Edinburgh Instrument, UK) equipped using a 980 nm laser as the excitation source. The Ultraviolet-Visible (UV–vis) absorption spectra were measured on a Perkin–Elmer Lambder 25 spectrophotometer.

## Additional Information

**How to cite this article**: Tian, B. *et al.* Construction of pH-responsive and up-conversion luminescent NaYF_4_:Yb^3+^/Er^3+^@SiO_2_@PMAA nanocomposite for colon targeted drug delivery. *Sci. Rep.* **6**, 21335; doi: 10.1038/srep21335 (2016).

## Supplementary Material

Supplementary Information

## Figures and Tables

**Table 1:** Parameters from curve fitting of drug release in different pH.

pH	n	K	R^2^
1.2	0.65 ± 0.04	12.24 ± 0.57	0.99
7.4	0.56 ± 0.04	20.94 ± 1.01	0.98
