# Supplementary material for: Construction of pH-responsive and up-conversion luminescent NaYF4:Yb3+/Er3+@SiO2@PMAA nanocomposite for colon targeted drug delivery
Source: Sci Rep. 2016 Feb 19;6:21335. doi: 10.1038/srep21335 (PMC4759527; doi:10.1038/srep21335)
Supplement: Supplementary Information [file srep21335-s1.pdf]

Supplementary Information

Construction of pH-responsive and up-conversion luminescent $NaYF_4:Yb^{3+}/Er^{3+}$@SiO_2@PMAA nanocomposite for colon targeted drug delivery

Boshi Tian[1], Shaohua Liu[1], Wei Lu[2], Lin Jin[1], Qingfeng, Li[1], Yurong, Shi[1], Chunyang Li[1], Zhenling Wang[1,]* and Yaping Du[3,]*

[1]The Key Laboratory of Rare Earth Functional Materials and Applications, Zhoukou Normal University, Zhoukou 466001, P. R. China.

[2]University Research Facility in Materials Characterization and Device Fabrication, T he Hong Kong Polytechnic University, Hong Kong, P. R. China.

[3]Frontier Institute of Science and Technology Jointly with College of Science, Xi'an Jiaotong University, Xi'an 710049, P. R. China.

---

*Corresponding authors. Tel. & Fax: +86-394-8178518.

E-mail address: zlwang2007@hotmail.com (Z. Wang), ypdu2013@mail.xjtu.edu.cn (Y. Du)

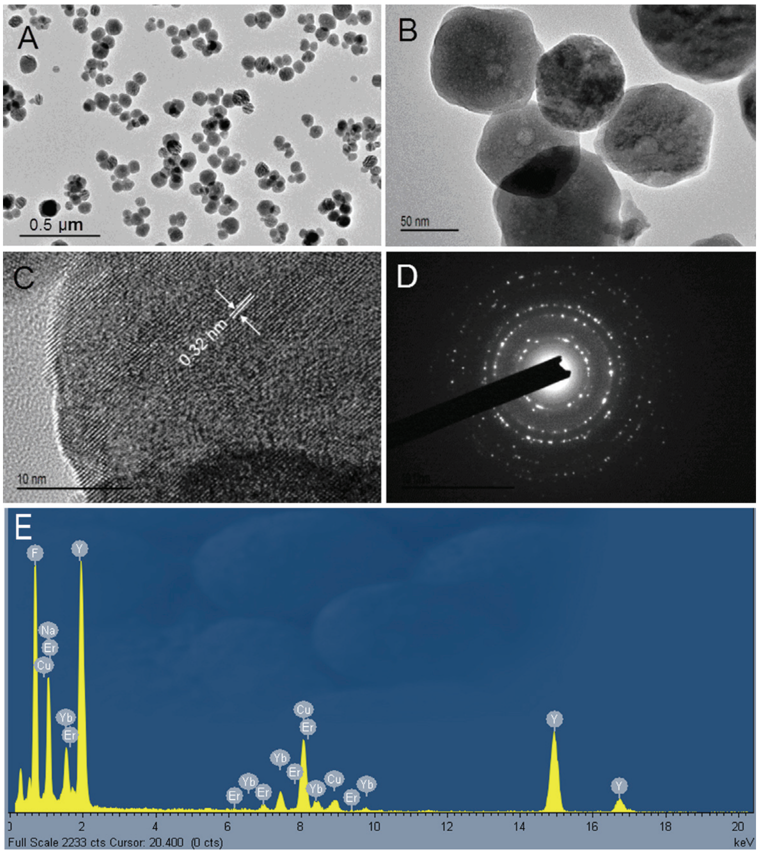

Figure S1. TEM images with low (A) and high (B) magnification, HRTEM (C), SAED pattern (D) and EDS spectrum (E) of UCNPs.

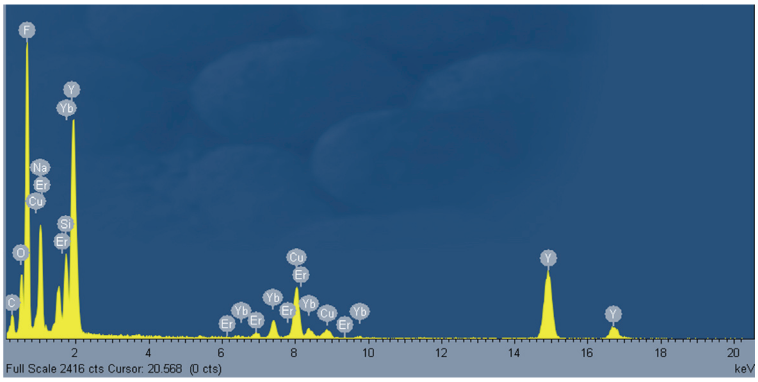

Figure S2. The energy dispersive X-ray spectroscopy (EDS) spectrum of UCNPs@SiO_2@PMAA.

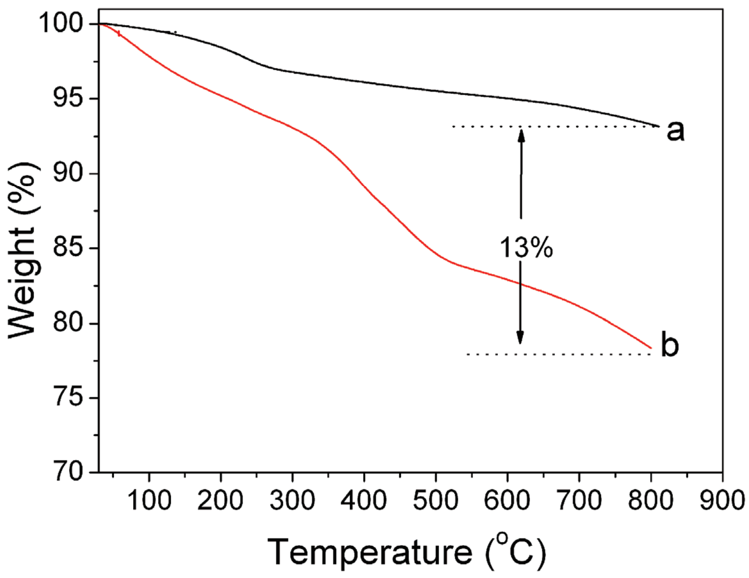

Figure S3. TGA curves of UCNPs@SiO_2-MPS (a) and UCNPs@SiO_2@PMAA (b).

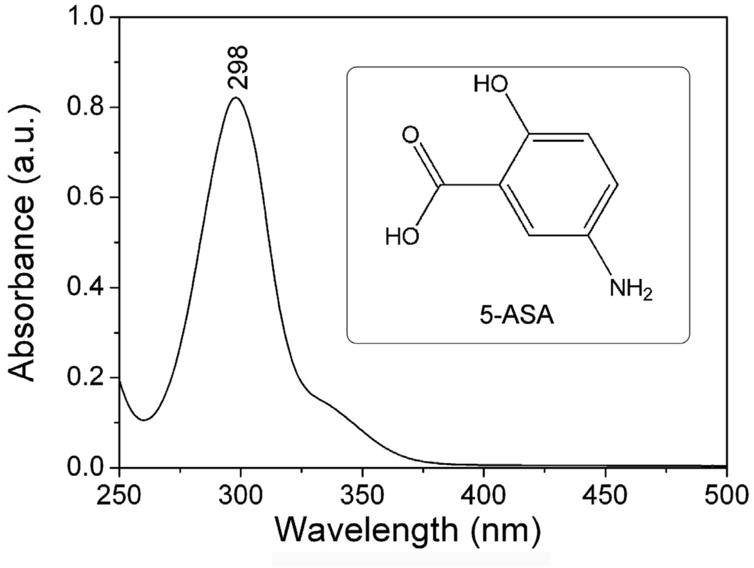

Figure S4. UV-vis spectrum of pure 5-ASA in PBS solution (pH = 7.4). Inset shows the structure of 5-ASA.

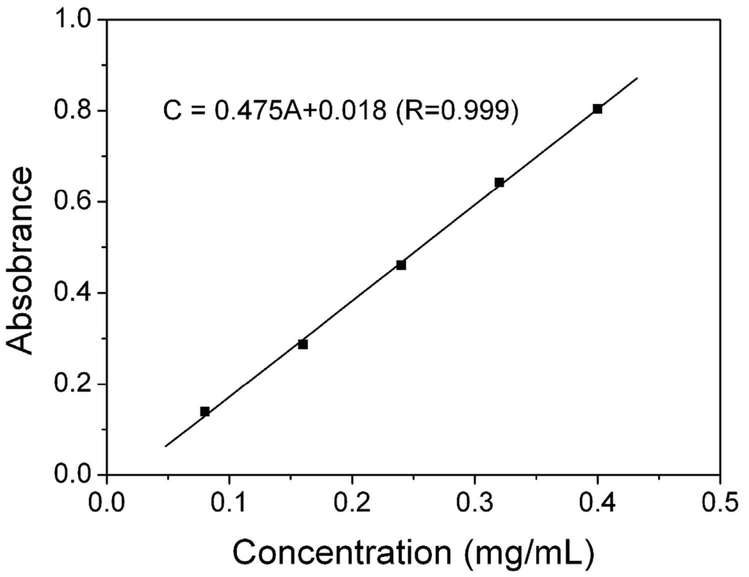

Figure S5. The standard curve of 5-ASA in PBS solution (pH = 7.4).
